# Clinical application of ultrasound-guided acupotomy combined with platelet-rich plasma in the treatment of carpal tunnel syndrome

**DOI:** 10.3389/fsurg.2025.1629781

**Published:** 2025-11-18

**Authors:** Guofei Ji, Wenxian Chen

**Affiliations:** Department of Ultrasound, Huzhou Central Hospital, The Fifth School of Clinical Medicine of Zhejiang Chinese Medical University, Huzhou, Zhejiang, China

**Keywords:** carpal tunnel syndrome, ultrasound, acupotomy, platelet-rich plasma, wrist joint function, median nerve conduction

## Abstract

**Background:**

This study aims to assess the impact of ultrasound-guided acupotomy combined with platelet-rich plasma (PRP) in treating carpal tunnel syndrome (CTS).

**Methods:**

A total of 72 CTS patients admitted to our hospital from June 2022 to December 2024 were divided into the control group and study group. The control group received ultrasound-guided acupotomy combined with dextrose 5% water treatment, while the study group received ultrasound-guided acupotomy combined with PRP therapy. The clinical efficacy, degree of pain, wrist joint function, median nerve conduction indicators, incidence of complications, and activities of daily living (ADL) were compared in both groups.

**Results:**

Compared with the control group, the study group had a higher total effective rate along with a lower incidence of complications (*P* < 0.05). Compared with before treatment, the visual analog scale (VAS), Boston carpal tunnel questionnaire (BCTQ)-symptom severity scale (SSS), and BCTQ-functional status scale (FSS) scores declined, and the ADL score was elevated in both groups at 1, 3, and 6 months following treatment (*P* < 0.05). Relative to the control group, the study group had lower VAS, BCTQ-SSS, and BCTQ-FSS scores and higher ADL scores at 1, 3, and 6 months following treatment (*P* < 0.05). Relative to before treatment, the values of sensory nerve conduction velocity (SNCV), motor nerve conduction velocity (MNCV), and complex muscle action potential (CMAP) amplitude were elevated in both groups following treatment (*P* < 0.05). Relative to the control group, the study group had higher values of SNCV, MNCV, and CMAP amplitude at 1, 3, and 6 months following treatment (*P* < 0.05). Relative to before treatment, the anterior–posterior diameter of the median nerve in the cross section of the hamate bone in both groups was longer after treatment (*P* < 0.05), and the transverse area of the median nerve in the cross section of the lenticular bone and the thickness of the transverse wrist ligament were smaller at 1, 3, and 6 months following treatment (*P* < 0.05). Nevertheless, there were no differences in the changes of the above ultrasound examination parameters between the two groups after treatment (*P* > 0.05).

**Conclusion:**

Ultrasound-guided acupotomy + PRP therapy has effective clinical efficacy in the treatment of CTS, which can alleviate the degree of pain, improve wrist joint function, promote the activities of daily living, reduce the incidence of complications, and improve the median nerve conduction.

## Introduction

Carpal tunnel syndrome (CTS) belongs to a common clinical ischemic disease caused by peripheral nerve entrapment (1). The main symptom of CTS is numbness and pain of the three and a half fingers of the radial side caused by median nerve entrapment, especially at night (2). With the extension of time, severe cases may exhibit muscle atrophy and hand dysfunction (3). The incidence of CTS ranges from 7% to 19%, and the incidence is higher in females than in males, which may be related to hormone levels in females (4). In recent years, with the popularity of mobile phones, computers, and other electronic devices, the incidence of CTS has increased year by year (5). Meanwhile, patients with diabetes, rheumatoid arthritis, hypothyroidism, and other systemic diseases are also high-risk factors for CTS (6). The treatment of CTS mainly includes conservative treatment and surgical release treatment (7). For early mild to moderate CTS, proper rest, change of working habits, and oral administration of neurotrophic drugs such as mecobalamine can improve symptoms (8). However, in real life, patients are often forced by the pressure of life, and the compliance with conservative treatment is poor, which often leads to the failure of traditional conservative treatment, and they are forced to undergo surgical treatment (9).

In recent years, ultrasound-guided minimally invasive therapy has been increasingly applied to CTS, and ultrasound-guided therapy has greatly improved the safety of treatment and reduced the complications of treatment (10). Ultrasound-guided steroid injection therapy can effectively improve the clinical symptoms of CTS in the short term, but the medium- and long-term prognosis is not ideal (11). Due to the extension of CTS over time, the median nerve often becomes adherent to the flexor tendon, and it is often difficult to achieve the purpose of loosening the adhesion by drug injection alone (12). At the same time, the cause of hyperplasia and thickening of the transverse ligament of the wrist is not relieved, and the recurrence of CTS is easy after intracarpal injection (13).

Musculoskeletal ultrasound is a new ultrasound technology emerging in recent years and has been applied more and more widely in the field of sports medicine (14). The peripheral nerve is relatively shallow, the bone structure is less occluded, and musculoskeletal ultrasound has high reliability in the diagnosis and differential diagnosis of peripheral nerve entrapment diseases (15). At the same time, after the cause of ultrasound is identified, the ultrasound-guided visualization operation technology, especially the visualization needle knife technology, can accurately guide the needle knife to the lesion site; implement accurate release treatment; avoid important tissues such as blood vessels, nerves, and tendons; and greatly reduce treatment complications (16).

Platelet-rich plasma (PRP) belongs to a regenerative medicine technology that has emerged in recent years (17). Through the extraction of autologous blood and centrifugation, a high concentration of platelet plasma is obtained (18). Platelets release a large number of growth factors required for repair during activation, promoting the repair of damaged tissues, and are increasingly used in the treatment of cartilage, tendon, ligament, and nerve injury (19). Especially in recent years, it has been used more and more in the repair treatment of nerve injury and has shown a remarkable therapeutic effect. Since PRP is prepared by using autologous blood, there is no immune rejection in theory, and it is safe, effective, and has broad clinical application prospects (20).

In our study, we aimed to explore the impacts of ultrasound-guided acupotomy plus PRP in the treatment of CTS.

## Methods

### General data

A total of 72 CTS patients admitted to our hospital from June 2022 to December 2024 were included as study participants and divided into the control group and study group. Each group consisted of 36 cases. The control group received ultrasound-guided acupotomy combined with dextrose 5% water (D5W) treatment, while the study group received ultrasound-guided acupotomy combined with PRP therapy.

Inclusion criteria: (1) patients with repeated chronic strain as the main cause; (2) patients with numbness, pain, and discomfort in the three and a half fingers of the radial side of the median nerve innervation area; (3) patients with night pain, numbness, and other effects on sleep; (4) the diagnosis met the diagnostic criteria for mild to moderate CTS; (5) patients aged ≥18 years old; (6) and patients who had not received any treatment in the past 2 weeks.

Exclusion criteria: (1) patients with rheumatoid, gout, and other rheumatic immune arthritis; (2) abnormal coagulation function; (3) puncture site infection; (4) patients with serious heart, liver, and kidney diseases and could not cooperate with and tolerate treatment; (5) wrist surgery history; (6) patients unable to complete treatment and follow-up; (7) patients who received anticoagulant therapy (such as aspirin, warfarin or heparin) 1 week before surgery; (8) pregnant woman; (9) platelet <125 × 10^9^/L; (10) patients who had taken a non-steroidal anti-inflammatory drug within 48 h; (11) patients who had used glucocorticoid hormones locally or systemic within 1 month.

### Randomization and blinding

A group randomization design was adopted for random grouping. The random allocation sequence was generated by a computer. The allocation confidentiality measures were achieved through sequential numbering, sealing, and opaque envelopes. After being deemed to meet the inclusion criteria, patients were randomly assigned to the control group or the study group in a 1:1 ratio. This study was single-blind, and the participants were unaware of the allocation.

### Sample size calculation

In this study, the degree of pain was selected as the main outcome variable for sample size calculation. Based on Cohen's proposed effect size criteria and the research experience in this field (21), we estimated that the ultrasound-guided acupotomy combined with PRP treatment would produce a moderate effect difference in pain level (VAS score) compared with the combined D5W treatment. We set the expected effect size (Cohen's *d*) at 0.5. The set parameters (the main outcome variable being the VAS score of pain level, the expected effect size being Cohen's *d* = 0.5, the test type being the two-independent-sample *t*-test, *α* = 0.05, 1 − *β* = 90%) were input into the G*Power 3.1.9.7 software for calculation. The results showed that at least 34 patients were required in each group to meet the statistical requirements of the study. Considering that unforeseen situations such as patient dropout and data loss may occur during the research process, to ensure the final effective sample size, we increased the dropout rate by 5%. After calculation, 36 patients were included in each group, and a total of 72 patients were included in the two groups.

### PRP preparation method

The centrifuge adopted by Jiangsu Changzhou Liangyou Medical Equipment Co., Ltd. (Model: TD5Z low-speed centrifuge) has a horizontal rotor. The anticoagulant was sodium citrate for blood transfusion produced by Tianjin Jinyao Pharmaceutical Co., Ltd., with specification: 10 mL: 0.25 g/ piece. Two milliliters of sodium citrate anticoagulant were extracted from a 20 mL screw syringe, and 18 mL of blood was collected from the patient's elbow vein into the syringe, mixed with the anticoagulant, and put into the centrifuge hanging cup. The corresponding volume of normal saline was added to the opposite hanging cup.

A two-step centrifugation procedure was adopted: 1,360 × *g* was used for the first centrifugation, for 10 min. The blood in the syringe tube was divided into three layers, and the lowest red blood cell layer was removed. The remaining serum was centrifuged again, at 1,360 × *g*, for 10 min, and then the poor platelet supernatant was removed. The remaining 2.0 mL was PRP.

### Treatment methods

The ultrasonic instrument adopted the Italian Baisheng Mylab90 color ultrasonic diagnostic instrument, electronic linear array probe, operating at a frequency of 9–12 MHz. The needle knife used was a Hanzhang No. 4 needle knife produced by Beijing Huaxia Needle Knife Medical Equipment Factory, with a diameter of 1.2 mm and a length of 50 mm.

Preoperative musculoskeletal ultrasonography was performed by an associate chief physician to determine whether the transverse ligament of the wrist was thickened; to evaluate the median nerve for signs of compression, edema, and thickening; and to determine the relative position of the transverse ligament of the wrist, the median nerve, and the ulnar artery. To measure the thickness of the flexor retinaculum, first, the ultrasound probe was placed parallel to the direction of the flexor tendon's course on the palmar side of the wrist, and a slow sliding scan was performed from the proximal end of the wrist to the distal end. During the scan, the shape, boundary, and internal echo of the wrist flexor tendon sheath were carefully observed. Based on the initial observation, the layer where the flexor tendon sheath was displayed most clearly and completely was selected for measurement. After determining the measurement layer, the position of the ultrasound probe was fixed to avoid measurement errors caused by the probe’s movement. The measurement tool of the ultrasound device was used to measure the thickness of the flexor tendon sheath on the cross section of the sheath. During the operation, the measurement cursor was placed on the inner and outer walls of the flexor tendon sheath, respectively, and the distance between the two points was measured, which was the thickness of the flexor retinaculum. To reduce measurement errors, we conducted three independent measurements on the same measurement layer and took the average value of the three measurements as the final thickness value of the flexor retinaculum for the patient.

#### Methods of ultrasound-guided acupotomy

The patient took the seated position and placed the wrist flat on the operating table, palm up, and fingers straight. In the surgical area of 5% povidone iodine, the median nerve, transverse carpal ligament, and ulnar artery were examined by musculoskeletal ultrasound. After planning the puncture path, the probe was placed on the longitudinal section, and the thickened transverse carpal ligament and median nerve were placed in the middle of the image. Under the guidance of ultrasound, the in-plane puncture technique was applied, and 1% lidocaine was used for local subcutaneous infiltration and anesthesia of the transverse ligament of the wrist. Then, under the guidance of ultrasound, the No. 4 Hanzhang needle knife was guided to the thickening of the transverse ligament of the wrist, and the flat knife was inserted into the needle. After the ultrasonic cross section and longitudinal section scan were combined to confirm the position of the needle knife, the knife was raised, and the transverse ligament of the wrist was relieved layer by layer, 3–5 times. The median nerve and ulnar artery were protected during the operation.

#### PRP treatment method

After the end of acupotomy treatment, the puncture needle was guided into the surface of the median nerve in the carpal canal under the guidance of ultrasound in the longitudinal section, and 2 mL PRP was injected around the median nerve in the carpal canal.

#### D5W treatment method

After the end of acupotomy treatment, the puncture needle was guided into the median nerve surface of the carpal canal under the ultrasonic guidance of the longitudinal section, and 2 mL 5% glucose was injected around the median nerve of the carpal canal.

### Observation indicators

1. Six months after treatment, the evaluation indexes of efficacy were developed according to the Kelly criteria (22). (1) Cure: The clinical symptoms disappeared completely, and the wrist function returned to normal. (2) Obvious effect: The clinical symptoms improved significantly, and most of the hand functions were restored. (3) Effective: The clinical symptoms improved, and hand function partially recovered. (4) Ineffective: The patient did not return to the above criteria. Total effective rate = (cure + obvious effect + effective)/total cases × 100%.
2. The visual analog scale of pain (VAS) was used for assessing the severity of pain before treatment and at 1, 3, and 6 months following treatment (23), with a score range of 0–10 points: 0 indicated no pain and 10 indicated the most unbearable pain. The higher the number, the greater the pain degree.
3. The wrist joint function was assessed by the Boston carpal tunnel questionnaire (BCTQ) before treatment and at 1, 3, and 6 months following treatment (24). The scale had two subscales: symptom severity scale (SSS) and functional status scale (FSS), which contained 11 symptom severity and eight functional status problems, with a score of 1–5 points for each item. The higher the score, the more severe the symptoms or the worse the function was.
4. Dantec Keypoint 9033A07 electromyography was used for examining median nerve conduction indicators, including sensory nerve conduction velocity (SNCV), motor nerve conduction velocity (MNCV), and complex muscle action potential (CMAP) amplitude before treatment and at 1, 3, and 6 months following treatment.
5. The anterior–posterior diameter of the median nerve in the cross section of the hamate bone, the transverse area of the median nerve in the cross section of the lenticular bone, and the thickness of the transverse wrist ligament were measured by ultrasound before treatment and at 1, 3, and 6 months following treatment.
6. Incidence of complications including hematoma, nerve injury, tendon injury, and wrist pain was recorded in both groups.
7. The activities daily living (ADL) scale was used for assessing activities of daily living (25), and the total score was 100 points. The higher the score, the stronger the activities of daily living of patients.

### Statistical analysis

SPSS 25.0 software was adopted for statistical analysis. The statistical data were expressed as rate, and the *χ*^2^ test was adopted for comparison. Measurement data were expressed as (*x* ± *s*), and *t*-test and repeated-measures ANOVA followed by *post hoc* comparisons were adopted for comparison. *P* < 0.05 meant the difference was significant.

## Results

### General data of patients in both groups

No significant difference was seen in the general data of patients between the two groups (*P* > 0.05, Table 1).

**Table 1:** General data of patients in both groups.

Groups	Cases	Gender		Age (years)	Course of disease (months)	Location of disease	
		Male	Female			Left hand	Right hand
Control group	36	11 (30.56)	25 (69.44)	45.43 ± 6.72	28.48 ± 13.82	15 (41.67)	21 (58.33)
Study group	36	12 (33.33)	24 (66.67)	45.51 ± 6.75	28.75 ± 14.36	14 (38.89)	22 (61.11)
*χ*^2^/*t*-value		0.06		0.05	0.08	0.05	
*P*-value		0.80		0.96	0.93	0.81	

### Clinical efficacy in both groups

Compared with the control group, the study group had a higher total effective rate 6 months following treatment (*P* < 0.05, Table 2).

**Table 2:** Clinical efficacy in both groups.

Groups	Cases	Cure	Obvious effect	Effective	Ineffective	Total effective rate
Control group	36	5 (13.90)	7 (19.44)	17 (47.22)	7 (19.44)	29 (80.56)
Study group	36	8 (22.22)	10 (27.78)	17 (47.22)	1 (2.78)	35 (97.22)
*χ*^2^ value						5.06
*P*-value						0.02

### Degree of pain in both groups

A repeated-measures analysis of variance was employed, with “group” (study group, control group) as the between-group factor and “time” (before treatment, 1 month after treatment, 3 months after treatment, 6 months after treatment) as the within-group factor, to analyze the VAS scores of the two groups of patients.

The time main effect was significant [*F* (1, 280) = 177.2, *P* < 0.001], the group main effect was significant [*F* (3, 280) = 723.9, *P* < 0.001], and the “group × time” interaction effect was significant [*F* (3, 280) = 23.08, *P* < 0.001].

The VAS scores of the study group at 1, 3, and 6 months after treatment were all significantly lower than those before treatment (all *P* < 0.001). Specifically, the VAS score before treatment was 6.51 ± 0.66, which decreased to 4.32 ± 0.43 1 month after treatment, 3.34 ± 0.35 3 months after treatment, and 2.52 ± 0.26 6 months after treatment.

The VAS scores of the control group at 1, 3, and 6 months after treatment were all significantly lower than those before treatment (all *P* < 0.001). Specifically, the VAS score before treatment was 6.49 ± 0.65, which decreased to 5.32 ± 0.53 1 month after treatment, 4.56 ± 0.46 3 months after treatment, and 3.32 ± 0.32 6 months after treatment.

**Figure 1:**
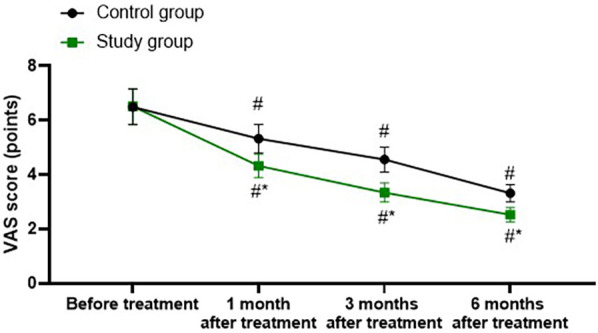
Degree of pain in both groups. ^#^*P* < 0.05, vs. before treatment; *P < 0.05, vs. control group.

One, 3, and 6 months after the treatment, the VAS score of the study group was significantly lower than that of the control group (all *P* < 0.001), and the specific data were the same as those at the corresponding time points mentioned above, as shown in Figure 1.

### Wrist joint function in both groups

A repeated-measures analysis of variance was employed, with “group” (study group, control group) as the between-group factor and “time” (before treatment, 1 month after treatment, 3 months after treatment, 6 months after treatment) as the within-group factor, to analyze the BCTQ-SSS score and BCTQ-FSS score of the two groups.

For BCTQ-SSS score, the time main effect was significant [*F* (1, 280) = 107.6, *P* < 0.001], the group main effect was significant [*F* (3, 280) = 703.4, *P* < 0.001], and the “group × time” interaction effect was significant [*F* (3, 280) = 13.23, *P* < 0.001].

The BCTQ-SSS scores of the study group at 1, 3, and 6 months after treatment were all significantly lower than those before treatment (all *P* < 0.001). Specifically, the BCTQ-SSS score before treatment was 35.68 ± 3.62, which decreased to 25.65 ± 2.54 1 month after treatment, 20.65 ± 2.06 3 months after treatment, and 13.28 ± 1.35 6 months after treatment.

The BCTQ-SSS scores of the control group at 1, 3, and 6 months after treatment were all significantly lower than those before treatment (all *P* < 0.001). Specifically, the BCTQ-SSS score before treatment was 35.65 ± 3.56, which decreased to 30.65 ± 3.62 1 month after treatment, 25.65 ± 2.54 3 months after treatment, and 16.85 ± 1.87 6 months after treatment.

**Figure 2:**
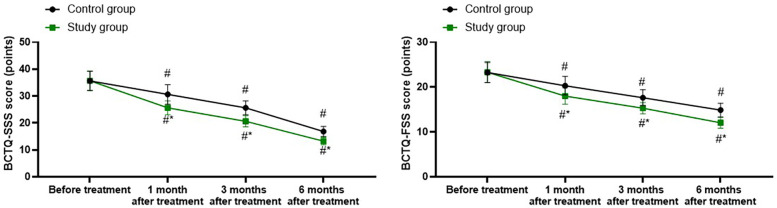
Wrist joint function in both groups. ^#^*P* < 0.05, vs. before treatment; *P < 0.05, vs. control group.

1, 3, and 6 months after the treatment, the BCTQ-SSS scores of the study group were significantly lower than those of the control group (all *P* < 0.001), and the specific data were the same as those at the corresponding time points mentioned above, as shown in Figure 2.

For BCTQ-FSS score, the time main effect was significant [*F* (1, 280) = 74.53, *P* < 0.001], the group main effect was significant [*F* (3, 280) = 378.0, *P* < 0.001], and the “group × time” interaction effect was significant [*F* (3, 280) = 9.254, *P* < 0.001].

The BCTQ-FSS scores of the study group at 1, 3, and 6 months after treatment were all significantly lower than those before treatment (all *P* < 0.001). Specifically, the BCTQ-FSS score before treatment was 23.32 ± 2.35, which decreased to 18.02 ± 1.81 1 month after treatment, 15.32 ± 1.25 3 months after treatment, and 12.05 ± 1.21 6 months after treatment.

The BCTQ-FSS scores of the control group at 1, 3, and 6 months after treatment were all significantly lower than those before treatment (all *P* < 0.001). Specifically, the BCTQ-FSS score before treatment was 23.25 ± 2.23, which decreased to 20.32 ± 2.06 1 month after treatment, 17.65 ± 1.76 3 months after treatment, and 14.89 ± 1.52 6 months after treatment.

One, 3, and 6 months after the treatment, the BCTQ-SSS scores of the study group were significantly lower than those of the control group (all *P* < 0.001), and the specific data were the same as those at the corresponding time points mentioned above, as shown in Figure 2.

### Electromyography examination parameters in both groups

A repeated-measures analysis of variance was employed, with “group” (study group, control group) as the between-group factor and “time” (before treatment, 1 month after treatment, 3 months after treatment, 6 months after treatment) as the within-group factor, to analyze the values of SNCV, MNCV, and CMAP amplitude of the two groups of patients.

For SNCV, the time main effect was significant [*F* (1, 280) = 100.8, *P* < 0.001], the group main effect was significant [*F* (3, 280) = 832.1, *P* < 0.001], and the “group × time” interaction effect was significant [*F* (3, 280) = 11.71, *P* < 0.001].

The SNCV of the study group at 1, 3, and 6 months after treatment were all significantly higher than those before treatment (all *P* < 0.001). Specifically, the SNCV before treatment was 17.61 ± 1.75, which increased to 30.52 ± 3.05 1 month after treatment, 40.28 ± 4.06 3 months after treatment, and 46.52 ± 4.65 6 months after treatment.

The SNCV of the control group at 1, 3, and 6 months after treatment were all significantly higher than those before treatment (all *P* < 0.001). Specifically, the SNCV before treatment was 17.65 ± 1.76, which increased to 25.65 ± 2.52 1 month after treatment, 35.14 ± 3.52 3 months after treatment, and 40.65 ± 4.16 6 months after treatment.

**Figure 3:**
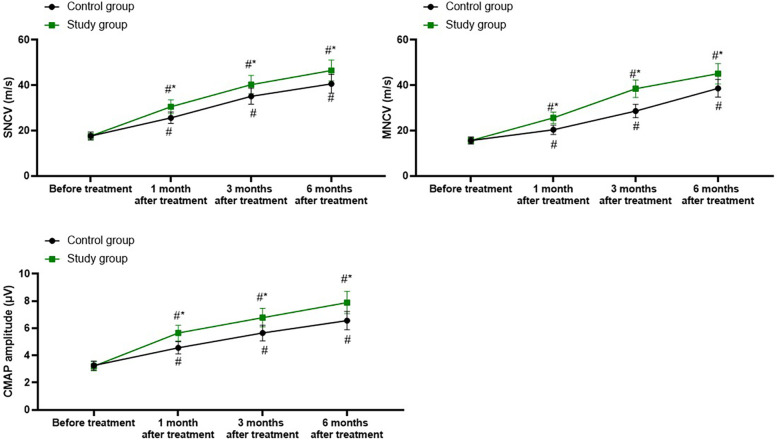
Electromyography examination parameters in both groups. ^#^*P* < 0.05, vs. before treatment; *P < 0.05, vs. control group.

One, 3, and 6 months after the treatment, the SNCV of the study group was significantly higher than those of the control group (all *P* < 0.001), and the specific data were the same as those at the corresponding time points mentioned above, as shown in Figure 3.

For MNCV, the time main effect was significant [*F* (1, 280) = 226.7, *P* < 0.001], the group main effect was significant [*F* (3, 280) = 1,046, *P* < 0.001], and the “group × time” interaction effect was significant [*F* (3, 280) = 32.61, *P* < 0.001].

The MNCV of the study group at 1, 3, and 6 months after treatment were all significantly higher than those before treatment (all *P* < 0.001). Specifically, the MNCV before treatment was 15.60 ± 1.51, which increased to 25.62 ± 2.57 1 month after treatment, 38.47 ± 3.85 3 months after treatment, and 45.12 ± 4.52 6 months after treatment.

The MNCV of the control group at 1, 3, and 6 months after treatment were all significantly higher than those before treatment (all *P* < 0.001). Specifically, the MNCV before treatment was 15.62 ± 1.56, which increased to 20.35 ± 2.03 1 month after treatment, 28.65 ± 2.86 3 months after treatment, and 38.65 ± 3.85 6 months after treatment.

One, 3, and 6 months after the treatment, the MNCV of the study group was significantly higher than that of the control group (all *P* < 0.001), and the specific data were the same as those at the corresponding time points mentioned above, as shown in Figure 3.

For CMAP, the time main effect was significant [*F* (1, 280) = 166.6, *P* < 0.001], the group main effect was significant [*F* (3, 280) = 635.4, *P* < 0.001], and the “group × time” interaction effect was significant [*F* (3, 280) = 21.28, *P* < 0.001].

The CMAP of the study group at 1, 3, and 6 months after treatment were all significantly higher than those before treatment (all *P* < 0.001). Specifically, the CMAP before treatment was 3.21 ± 0.33, which increased to 5.65 ± 0.57 1 month after treatment, 6.78 ± 0.68 3 months after treatment, and 7.89 ± 0.82 6 months after treatment.

The CMAP of the control group at 1, 3, and 6 months after treatment were all significantly higher than those before treatment (all *P* < 0.001). Specifically, the CMAP before treatment was 3.26 ± 0.32, which increased to 4.56 ± 0.45 1 month after treatment, 5.65 ± 0.57 3 months after treatment, and 6.56 ± 0.67 6 months after treatment.

One, 3, and 6 months after the treatment, the CMAP of the study group was significantly higher than that of the control group (all *P* < 0.001), and the specific data were the same as those at the corresponding time points mentioned above, as shown in Figure 3.

### Ultrasound examination parameters in both groups

**Figure 4:**
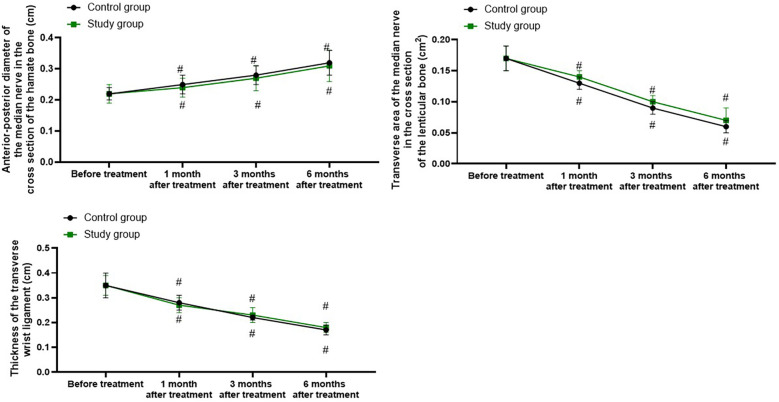
Ultrasound examination parameters in both groups. ^#^*P* < 0.05, vs. before treatment.

Relative to before treatment, the anterior–posterior diameter of the median nerve in the cross section of the hamate bone in both groups was longer at 1, 3, and 6 months following treatment (*P* < 0.05), and the transverse area of the median nerve in the cross section of the lenticular bone and the thickness of the transverse wrist ligament were smaller at 1, 3, and 6 months following treatment (*P* < 0.05). Nevertheless, there were no differences in the changes of the above ultrasound examination parameters between the two groups at 1, 3, and 6 months following treatment (*P* > 0.05, Figure 4).

### Incidence of complications in both groups

Relative to the control group, the study group had a lower incidence of complications (*P* < 0.05, Table 3).

**Table 3:** Incidence of complications in both groups.

Groups	Cases	Hematoma	Nerve injury	Tendon injury	Wrist pain	Total incidence rate
Control group	36	3 (8.33)	2 (5.56)	2 (5.56)	3 (8.33)	10 (27.78)
Study group	36	1 (2.78)	1 (2.78)	1 (2.78)	0 (0.00)	3 (8.34)
*χ*^2^ value						4.60
*P*-value						0.03

### Activities of daily living in both groups

A repeated-measures analysis of variance was employed, with “group” (study group, control group) as the between-group factor and “time” (before treatment, 1 month after treatment, 3 months after treatment, 6 months after treatment) as the within-group factor, to analyze the ADL score of the two groups of patients.

The time main effect was significant [*F* (1, 280) = 163.5, *P* < 0.001], the group main effect was significant [*F* (3, 280) = 1,108, *P* < 0.001], and the “group × time” interaction effect was significant [*F* (3, 280) = 20.5, *P* < 0.001].

The ADL scores of the study group at 1, 3, and 6 months after treatment were all significantly higher than those before treatment (all *P* < 0.001). Specifically, the ADL score before treatment was 29.00 ± 2.89, which increased to 60.65 ± 6.05 1 month after treatment, 75.65 ± 7.52 3 months after treatment, and 91.28 ± 8.52 6 months after treatment.

The ADL scores of the control group at 1, 3, and 6 months after treatment were all significantly higher than those before treatment (all *P* < 0.001). Specifically, the ADL score before treatment was 29.05 ± 2.93, which increased to 45.65 ± 4.56 1 month after treatment, 62.85 ± 6.32 3 months after treatment, and 81.52 ± 8.15 6 months after treatment.

**Figure 5:**
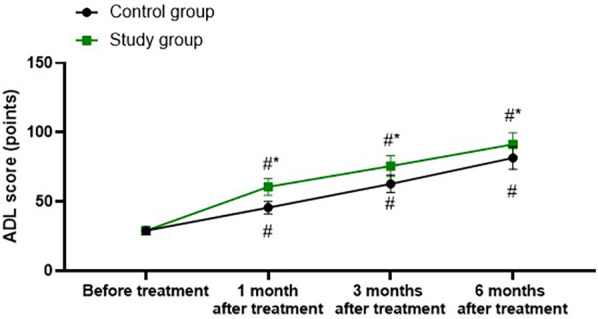
Activities of daily living in both groups. ^#^*P* < 0.05, vs. before treatment; *P < 0.05, vs. control group.

One, 3, and 6 months after the treatment, the ADL score of the study group was significantly higher than that of the control group (all *P* < 0.001), and the specific data were the same as those at the corresponding time points mentioned above, as shown in Figure 5.

## Discussion

At present, the clinical treatment of CTS is aimed at relieving the compression of the median nerve in the carpal canal, improving the degree of nerve damage, hand and wrist symptoms, and paresthesia (26). Surgery is the main method for the treatment of CTS, such as endoscopic surgery and minimally invasive surgery, which are widely applied in treating CTS, and the therapeutic effect is relatively ideal (27). Moreover, with the maturity of ultrasonic visualization technology, the structure of the carpal canal can be observed in real time and dynamically, especially the nerves and blood vessels in the carpal canal, which has the advantages of safety, convenience, and no radiation (28). However, surgical treatment has certain trauma, postoperative complications are easy to occur, and the surgical cost is high (29). With the development of medical technology, acupotomy has emerged as a new method for the treatment of CTS (30). Acupotomy can release the diseased tissue and fascia, improve the local blood circulation, promote the regeneration of local tissue, restore the dynamic balance of the diseased area, and realize the effective treatment of CTS (21).

In addition, median nerve block in the carpal canal is also an effective treatment for CTS (31). D5W was initially reported to be used as an isolating fluid to isolate tissue during nerve block, and it was accidentally found that it can change the nerve block effect after local anesthesia, and it has been increasingly used in peripheral nerve entrapment therapy (32). Research reports on D5W in the treatment of CTS have gradually increased in recent years. Studies have shown that compared with splintage and steroid injection, D5W can improve the pain, nerve electrophysiological changes, and median nerve cross-sectional area of patients 6 months after treatment, and the incidence of complications is lower (33, 34). The specific mechanism of D5W in the treatment of CTS has not been clarified, which may be related to its repression of transient receptor potential vanilloid receptor-1 (TRPV1) and the reduction of pain factors secreted by swollen nerves (35). However, due to the mild effect of D5W, slow onset, the specific mechanism is unknown, and the clinical application is not much.

Nerve regeneration after peripheral nerve injury is a very complex process, and the local microenvironment plays a very important role (36). Rebuilding the microenvironment of nerve regeneration will help protect the damaged neurons and promote the growth and functional recovery of axons (37). A large number of experiments have shown that the introduction of exogenous growth factors into the damaged local microenvironment at the early stage of peripheral nerve injury is conducive to nerve regeneration and repair and reduces the incidence of postoperative complications (38).

PRP is a concentrated platelet plasma that releases a lot of growth factors during platelet activation, including platelet-derived growth factor, transforming growth factor, fibroblast growth factor, insulin-like growth factor-1, insulin-like growth factor-2, vascular endothelial growth factor, epidermal growth factor, interleukin-8, keratinocyte growth factor, and connective tissue growth factor (39). PRP interacts with growth factors to activate target protein receptors and induce protein regeneration; thus, promoting cell proliferation, collagen formation, and the repair of human tissues (40). In recent years, PRP has been applied more and more in the treatment of nerve injury repair, and a large number of growth factors released by PRP have shown a remarkable therapeutic effect in the field of nerve injury repair (41). PRP promotes vascular regeneration and axon regeneration of ischemic nerve, with high safety, good effect, and no immune rejection, especially in the therapy of peripheral neuropathy (42). A randomized controlled study on CTS showed that the injection of PRP into the carpal canal under ultrasound guidance can not only relieve the swelling and inflammation of the flexural tendon in the carpal canal, but also alleviate the symptoms of median nerve compression (43). At the same time, PRP can also widely act on the median nerve, promote the repair of median nerve ischemic injury, and improve sensory and motor function (20).

In this study, the clinical efficacy of ultrasound-guided acupotomy + D5W treatment and ultrasound-guided acupotomy + PRP therapy in CTS patients was compared. The results indicated that compared with the control group, the study group had a higher total effective rate along with a lower incidence of complications, suggesting that ultrasound-guided acupotomy + PRP therapy had better clinical efficacy in the treatment of CTS, and could effectively reduce the incidence of complications. Similarly, Catapano et al. (44) suggested that PRP represented a promising therapy for patients with mild to moderate CTS. Malahias et al. (45) performed a systematic and comprehensive review and indicated that PRP infusion improved the clinical condition of the patients and that PRP infusion was beneficial for patients with mild to moderate CTS.

Moreover, our study indicated that compared with before treatment, the VAS score, BCTQ-SSS score, and BCTQ-FSS score declined and the ADL score was elevated in both groups at 1, 3, and 6 months following treatment. Relative to the control group, the study group had lower VAS score, BCTQ-SSS score, and BCTQ-FSS score as well as higher ADL score at 1, 3, and 6 months following treatment. All these results suggested that ultrasound-guided acupotomy + PRP therapy could better reduce the degree of pain, improve wrist joint function, and promote the activities of daily living in the treatment of CTS. This is because in addition to having a positive effect on the nerves themselves, PRP may also have beneficial effects on other tissues within the wrist canal, such as tendons and synovium. It can regulate the local inflammatory response, reduce the stimulation and damage to the nerves caused by inflammatory mediators, improve local blood circulation, provide more sufficient nutrients and oxygen to the nerves and other tissues, and promote tissue repair and regeneration. This comprehensive improvement of the local microenvironment may help achieve a more comprehensive recovery of hand function and thus show better results in clinical evaluations. In contrast, D5W, although it can also improve the local environment to a certain extent, may not be able to have the synergistic regulatory effect on multiple tissues such as PRP, and therefore is relatively weaker in terms of functional improvement. Consistently, Gao et al. (46) indicated that PRP injection was the most likely to relieve symptoms, improve functions, and reduce pain among the injections of corticosteroid, D5W, and PRP. Dong et al. (47) conducted a systematic review and meta-analysis of randomized controlled trials and indicated that the PRP could be effective for mild to moderate CTS and superior to traditional conservative treatments in improving pain and function and reducing the swelling of the median nerve for a mid-long-term effect. To some extent, the electrophysiological indexes also improved after PRP injection compared with other conservative treatments.

Moreover, our study manifested that compared with before treatment, the values of SNCV, MNCV, and CMAP amplitude were elevated in both groups at 1, 3, and 6 months following treatment. Relative to the control group, the study group had higher values of SNCV, MNCV, and CMAP amplitude at 1, 3, and 6 months following treatment. Meanwhile, compared with before treatment, the anterior–posterior diameter of the median nerve in the cross section of the hamate bone in both groups was longer at 1, 3, and 6 months following treatment, and the transverse area of the median nerve in the cross section of the lenticular bone and the thickness of the transverse wrist ligament were smaller at 1, 3, and 6 months following treatment. However, there were no differences in the changes of the above ultrasound examination parameters between the two groups at 1, 3, and 6 months following treatment. All these results suggested that ultrasound-guided acupotomy + PRP therapy could better improve the median nerve conduction in the treatment of CTS. The ultrasound examination parameters and electrophysiological examination indicators used in this study are all commonly employed methods for evaluating the therapeutic effect of CTS. However, each of these indicators has its own limitations. Ultrasound examination mainly reflects the morphological changes of the nerves, but it may not be sensitive enough for subtle changes in nerve function. While electrophysiological examination can well reflect the state of nerve function, it cannot fully cover all aspects of hand function. The recovery of hand function is a multidimensional process involving multiple aspects such as sensation, movement, and coordination. The current assessment indicators may not be able to comprehensively and accurately reflect such complex functional changes. Therefore, the lack of correlation between structural changes and functional improvement may be due to the limitations of the existing assessment indicators, resulting in the failure to fully reveal the intrinsic relationship between the two. In line with our findings, Chen et al. (48) suggested that relative to the control group, the PRP group presented significant improvements in the cross-sectional area of the median nerve and electrophysiological study at the 12th month postinjection. Wu et al. performed a prospective randomized, single-blind, controlled trial and indicated that PRP is a safe modality that effectively relieves pain and improves median nerve function in patients with CTS (49).

Recently, ultrasound-guided hydrodissection has been widely used in the treatment of CTS (50). The technique mainly involves using ultrasound guidance to inject specific liquids (such as normal saline and glucocorticoids) into the carpal tunnel. The pressure of the liquid separates the adhesions and expands the space of the carpal tunnel, thereby reducing the compression on the nerves. Its mechanism is relatively simple and mainly focuses on alleviating nerve compression through physical means. Its effect on nerve repair and regeneration is relatively limited (51). Although some studies have also mentioned that the components in the liquid may have certain anti-inflammatory effects, compared with the abundant growth factors in PRP, its overall effect in promoting tissue repair and nerve function recovery is weaker (52).

An important limitation of our study design is the selection of D5W as the control substance. While we sought to match the physical properties of the injectate, it is recognized that perineural D5W injection may itself have neuromodulatory effects, potentially acting as a neurolytic agent on nociceptive fibers (53, 54). Consequently, our findings should be interpreted as evidence that PRP provides a significant added benefit to acupotomy beyond the established effects of D5W hydrodissection. This choice of active comparator likely results in a conservative estimate of the efficacy of PRP. Future randomized trials should include a saline placebo control to isolate the specific biological effect of PRP. Our research has some limitations. First, our sample size is relatively small, which may lead to deviations between the data results and the actual values. Second, our research adopted a single-blind design, which inevitably resulted in subjective biases from the researchers, leading to an imbalance in the treatment between the two groups. Third, our research was a single-center study, and the sample was not representative, which may not accurately reflect the characteristics of a broader population. Fourth, our research only conducted 6-month follow-up observations. The effects of ultrasound-guided acupotomy + PRP therapy on the long-term efficacy and wrist joint function of patients with CTS are currently unclear. Therefore, more multicenter, double-blind, large-scale, and long-term studies should be conducted in the future to further verify our findings.

## Conclusion

Our study demonstrates that ultrasound-guided acupotomy + PRP therapy has effective clinical efficacy in the treatment of CTS, which can decrease the degree of pain, improve wrist joint function, promote the activities of daily living, reduce the incidence of complications, and improve the median nerve conduction.

## Data Availability

The original contributions presented in the study are included in the article/Supplementary Material, further inquiries can be directed to the corresponding author.
